# The use of feature tracking to assess ventricular strain during exercise stress CMR

**DOI:** 10.1186/1532-429X-14-S1-P230

**Published:** 2012-02-01

**Authors:** Kaleab N Asrress, Rupert Williams, Tim Lockie, Dirk Lossnitzer, Kalpa De Silva, Roy Jogiya, Sebastian Kozerke, Phil Chowienczyk, Gerald F Greil, Michael Marber, Eike Nagel, Simon Redwood, Sven Plein

**Affiliations:** 1 Cardiovascular Sciences, King's College London, London, UK; 2 Imaging Sciences, King's College London, London, UK; 3 Multidisciplinary Cardiovascular Research Centre, University of Leeds, Leeds, UK

## Background

Exercise stress testing is the most physiological method of inducing myocardial stress, but its application to clinical and scientific CMR has been limited because of problems arising from cardiac and respiratory motion during stress. Under pharmacological stress, left ventricular strain measurement abnormalities by CMR have been demonstrated to be earlier and more sensitive markers of contractile dysfunction than global left ventricular ejection fraction or development of regional wall motion abnormalities alone. There are several validated methods of measuring myocardial strain. A recently proposed method is feature tracking (FT), which has advantages over other methods in having shorter acquisition and analysis times and not requiring additional scanning as the features are tracked from the clinically standard cine steady-state free precession (SSFP) sequences. This offers a potential method to assess myocardial strain during exercise stress.

## Methods

Seven healthy volunteers without known cardiovascular disease gave informed consent and enrolled for supine cycle ergometry on the CMR scanner table (Lode, Groningen Netherlands). Subjects underwent a standardised incremental exercise protocol. Imaging included a standard short-axis stack of cine SSFP images from the cardiac base to apex, on a 3T Philips Achieva TX® system using a 32-channel multi-transmit coil. Scan parameters included slice thickness of 10mm, repetition/echo time of 2.4/1.21ms, flip angle 40°, 9-11 slices, with 20 phases per slice.

For this study, only the mid LV-slice was analysed. Diogenes CMR FT software (TomTec Imaging Systems, Munich, Germany) was used for strain analysis. This was based on contours manually drawn along the LV endocardial border of one frame, with the software automatically propagating the contour and following its features throughout the remainder of the imaged phases. Software-derived parameters include circumferential epicardial and endocardial, longitudinal, and radial tissue velocity, strain, strain rate and time to peak strain.

## Results

Seven subjects (average age 39.4 years) underwent exercise stress. Table 1 shows the measured strain parameters. Mean circumferential endocardial and epicardial strain were increased significantly with exercise, as were strain rate (p < 0.05 for all). Time to peak strain was reduced (p < 0.05). Radial strain showed no difference between rest and stress, though strain rate and time to peak strain showed similar patterns to circumferential parameters.

**Table 1:** Summary of Feature Tracking parameters at rest and exercise stress. Data are presented as mean ±SEM. A p value of < 0.05 was considered significant.

Circumferential	Rest	Exercise	P Value
Endocardia Strain %	-19.2 ± 2.3	- 24.9 ± 2.8	0.047
Peak Strain Rate s-1	1.2 ± 0.2	2.0 ± 0.2	0.009
Time to peak strain ms	324 ± 12	248 ± 9	0.001
Endocardia Strain %	-11.9 ± 1.6	-15.2 ± 1.6	0.045
Peak Strain Rate s-1	0.7 ± 0.1	1.4 ± 0.2	0.002
Time to peak strain ms	335 ±13	249 ± 8	0.001
Radial			
Strain %	20.7 ± 2.2	24.3 ± 2.5	0.150
Peak Strain Rate s-1	1.1 ± 0.1	1.8 ± 0.1	0.002
Time to peak strain ms	299 ± 14	236 ± 7	0.005

## Conclusions

Feature tracking can be used to quantify circumferential and radial strain during cycle ergometer exercise stress in healthy volunteers. It provides a potential method for assessing deformation parameters in patients with coronary as well as structural heart disease.

## Funding

The authors are supported by Clinical Research Training Fellowships from the British Heart Foundation; as well as the Department of Health via the National Institute for Health Research comprehensive Biomedical Research Centre award to Guy’s and St Thomas’ NHS Foundation Trust.

